# Altered Cerebellar Functional Connectivity with Intrinsic Connectivity Networks in Adults with Major Depressive Disorder

**DOI:** 10.1371/journal.pone.0039516

**Published:** 2012-06-18

**Authors:** Li Liu, Ling-Li Zeng, Yaming Li, Qiongmin Ma, Baojuan Li, Hui Shen, Dewen Hu

**Affiliations:** 1 Department of Psychiatry, First Affiliated Hospital, China Medical University, Shenyang, Liaoning, China; 2 College of Mechatronics and Automation, National University of Defense Technology, Changsha, Hunan, China; 3 Department of Nuclear Medicine, First Affiliated Hospital, China Medical University, Shenyang, Liaoning, China; Hangzhou Normal University, China

## Abstract

**Background:**

Numerous studies have demonstrated the higher-order functions of the cerebellum, including emotion regulation and cognitive processing, and have indicated that the cerebellum should therefore be included in the pathophysiological models of major depressive disorder. The aim of this study was to compare the resting-state functional connectivity of the cerebellum in adults with major depression and healthy controls.

**Methods:**

Twenty adults with major depression and 20 gender-, age-, and education-matched controls were investigated using seed-based resting-state functional connectivity magnetic resonance imaging.

**Results:**

Compared with the controls, depressed patients showed significantly increased functional connectivity between the cerebellum and the temporal poles. However, significantly reduced cerebellar functional connectivity was observed in the patient group in relation to both the default-mode network, mainly including the ventromedial prefrontal cortex and the posterior cingulate cortex/precuneus, and the executive control network, mainly including the superior frontal cortex and orbitofrontal cortex. Moreover, the Hamilton Depression Rating Scale score was negatively correlated with the functional connectivity between the bilateral Lobule VIIb and the right superior frontal gyrus in depressed patients.

**Conclusions:**

This study demonstrated increased cerebellar coupling with the temporal poles and reduced coupling with the regions in the default-mode and executive control networks in adults with major depression. These differences between patients and controls could be associated with the emotional disturbances and cognitive control function deficits that accompany major depression. Aberrant cerebellar connectivity during major depression may also imply a substantial role for the cerebellum in the pathophysiological models of depression.

## Introduction

Major depressive disorder is a common mental disorder that is characterized by mood dysregulation and cognitive impairment [1]. Recently, increased attention has been placed on the higher-order functions of the cerebellum [2], including cognitive processing, emotional control, as well as learning and memory [3], [4], [5], [6], [7], [8], [9], [10], rather than the pure motor functions. Recent findings highlight the relevance of a functionally intact cerebellum-related network in neuropsychiatric patients. For instance, some previous studies have demonstrated abnormal cerebellar activities and connectivities in patients with major depression using task-related fMRI [11], [12], [13], [14]. During rest, significantly lower regional homogeneity in the cerebellum was also observed in depressed patients [15], [16], and impairments of the neural activities in the cerebellum were suggested to partially underlay the emotional and cognitive symptoms observed in depressed patients [16]. Additionally, the cerebellum has been previously identified as part of a functional network subserving executive processes in depression [17]. These findings not only provide valuable insights into the pathological mechanisms of this complex mental disorder but also indicate that the functioning of the cerebellum in neuropsychiatric patients could be more important than previously thought.

It has been proposed that major depressive symptoms are associated with dysregulation of a distributed neuronal network encompassing cortical and limbic regions rather than with the (functional) breakdown of a single discrete brain region [18], [19], [20], [21]. Recently, resting-state functional connectivity magnetic resonance imaging (rs-fcMRI) has attracted attention for its application to mapping large-scale neural network function and dysfunction [22]. The tonic nature of the core depression symptoms indicates that rs-fcMRI may be helpful for advancing the understanding of pathophysiological mechanisms underlying affective and cognitive dysfunctions in major depression [23], [24], [25]. Previous rs-fcMRI studies have detected intrinsic connectivity network alterations in patients with major depression, especially abnormalities in the default-mode network (DMN) and executive control network (ECN) [25], [26], [27], [28]. A recent study found altered cerebellar-cerebral functional connectivity in geriatric depression [29]. To date, cerebellar functional connectivity has not been systematically explored during rest in younger patients with major depression.

All of these findings together contribute to the hypothesis that the cerebellum may play a non-negligible role in the alterations of intrinsic connectivity networks, including areas known to be related to affective and cognitive processing, in adults with major depression. In the present study, we used seed-based rs-fcMRI to investigate differences in the cerebellar resting-state functional connectivity between depressed adults and healthy controls.

## Materials and Methods

### Subjects

The study's participants included 20 adult patients diagnosed with major depressive disorder from the outpatient clinic at the First Affiliated Hospital of China Medical University and 20 demographically similar healthy volunteers recruited via advertisements (Table 1). No subjects were removed due to excessive motion (>2.5 mm translation and >2° rotation). Correlation analysis is sensitive to gross head motion effects [30], [31], so we further characterized the mean displacement as a measure of head motion for each subject [30], [32] (Table 1). All of the subjects were right-handed native Chinese speakers. Depressed patients met the criteria for a current episode of unipolar recurrent major depression based on the DSM (Diagnostic and Statistical Manual of Mental Disorders)-IV criteria [1]. Using the Structured Clinical Interview for DSM-IV [33], confirmation of the diagnosis was made by clinical psychiatrists. All patients were medication-naive at the time of the scan. Exclusion criteria included acute physical illness, substance abuse or dependence, a history of head injury resulting in loss of consciousness, and major psychiatric or neurological illness other than depression. Similar exclusion criteria were adopted for healthy control subjects. On the days of the scans, the depressive symptoms of the patients were assessed by clinical psychiatrists using the 17-item Hamilton Depression Rating Scale (HDRS) [34] and Clinical Global Impression Scale-Severity (CGI-S) [35] (Table 1). Healthy volunteers were studied under identical conditions, and their depressive symptoms were also assessed by clinical psychiatrists using the 17-item HDRS on the day of scanning. This study was approved by the Ethics Committee of China Medical University, and all participants gave written informed consent.

**Table 1:** Characteristics of the participants in this study.

Variable	Patient	Control	*p*-value
Sample size	20	20	
Gender (M/F)	6/14	4/16	0.47^a^
Age (years)	28.4±8.2	28.95±6.92	0.82^b^
Education (years)	11.95±3.32	11.65±3.08	0.77^b^
Weight (kg)	60.75±11.3	61±9.35	0.94^b^
Number of previous episodes	1.65±0.81		
Duration of current episode (months)	5.50±6.84		
Hamilton Depression Rating Scale (HDRS)	26.1±5.0	4.05±0.97	
Clinical Global Impression Scale-Severity (CGI-S)	6.0±0.63		
Mean displacement (mm)	0.43±0.29	0.23±0.16	0.01^ b^

a Pearson Chi-square test; ^b^Two-sample t-test.

### Resting experiment and MRI image acquisition

In the experiments, subjects were simply instructed to keep their eyes closed, relax, remain awake, and perform no specific cognitive exercise. Magnetic resonance images were acquired using a 1.5-T GE SIGNA scanner (GE Medical Systems). To reduce head movement, the subjects' heads were fixed using foam pads with a standard birdcage head coil. All fMRI images were collected using a gradient-echo EPI sequence. The imaging parameters were as follows: repetition time/echo time (TR/TE)  = 2000/50 ms, thickness/gap  = 5/1.5 mm, field of view (FOV)  = 240×240 mm, flip angle (FA)  = 90°, matrix  = 64×64, and slices  = 20. Each functional resting-state session lasted ∼8 min, and 245 volumes were obtained.

### Data preprocessing

Resting-state fMRI images were preprocessed using SPM8 (http://www.fil.ion.ucl.ac.uk/spm). For each subject, the first five volumes of the scanned data were discarded for magnetic saturation. The remaining 240 volumes were corrected by registering and reslicing to account for head motion. Next, the volumes were normalized to the standard EPI template in the Montreal Neurological Institute space. The resulting images were spatially smoothed with a Gaussian filter of 8-mm full-width half-maximum kernel, detrended to abandon linear trend, and then temporally filtered with a band-pass filter (0.01–0.08 Hz). In addition to the regression of head motion parameters, whole brain signal, and ventricular signal, the white matter signal was also implemented to reduce spurious variance unlikely to reflect neuronal activity. To further reduce the negative impacts of those artifacts related to motion and physiological sources (especially greater head movement in the patient group) on the functional connectivity analysis, the time courses of noise components extracted using group independent component analysis (ICA) were utilized for artifact removal for each subject [36], [37]. The residuals of these regressions constituted the set of time series used for functional connectivity analysis.

### Selection of regions of interest (ROIs)

**Figure 1:**
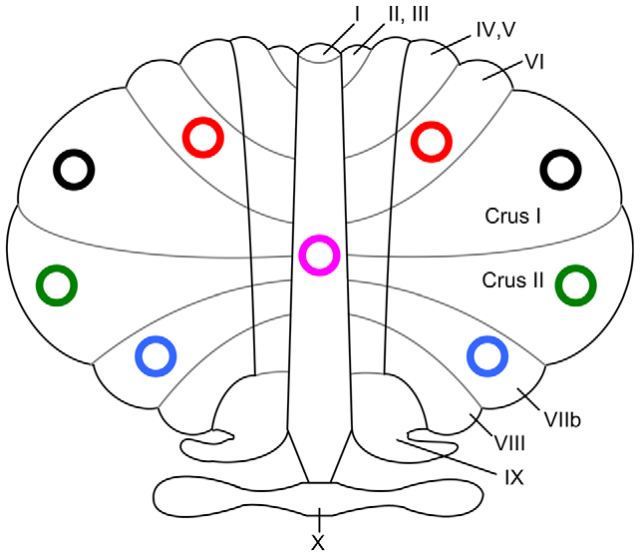
Schematic illustration of the regions of interest (ROIs). Two open circles with the same color represent both hemispheres of the corresponding ROI, respectively: Lobule VI (red), Crus I (black), Crus II (green), Lobule VIIb (blue), and the Vermis (pink).

A seed-based method was applied to identify differences in the resting-state functional connectivity involving the cerebellum between adults with major depression and healthy controls. For this purpose, several ROIs, including the cerebellar Lobule VI, Crus I, Crus II, Lobule VIIb, and Vermis, were defined a priori, as shown in Figure 1. Lobule VI, Crus I, Crus II, Lobule VIIb, and Vermis have been documented to participate in emotional and cognitive functioning [10], [38], [39], [40]. These ROIs have also been found to contribute to intrinsic connectivity networks in healthy subjects, including those involving the DMN, ECN, and salience network [4], [6], [39], [40]. As major depression is characterized by emotion dysregulation and cognitive impairments, and as simultaneous abnormalities in intrinsic connectivity networks have been found in previous studies [25], [27], we selected the bilateral Lobule VI, bilateral Crus I, bilateral Crus II, bilateral Lobule VIIb, and Vermis as the ROIs in this study. All ROI masks were generated using the free software WFU_PickAtlas (http://www.ansir.wfubmc.edu).

### Functional connectivity analysis

Regional mean time series were obtained for each individual by averaging the fMRI time series over all voxels in each seed region. We evaluated functional connectivity between each pair of time series using the Pearson correlation coefficient. Then, functional connectivity maps were produced by computing the correlation coefficients between each ROI signal and the time series from all other voxels within the brain [41]. In addition, all correlation coefficients were converted to *z*-scores by applying the Fisher *r*-to-*z* transformation.

### Statistical analysis and clinical correlation analysis

To explore any differences between the patient and healthy groups with regard to cerebellar connectivity with all other brain voxels, a second-level random-effect two-sample t-test was performed in SPM8 on the individual z-score connectivity maps in a voxel-by-voxel manner. The resulting statistical maps were set at a combined threshold of *p*<0.05 (False Discovery Rate corrected) with cluster size >10 voxels. For the maps which did not meet this criterion, a relaxed threshold of *p*<0.001 (uncorrected) with cluster size >50 voxels was also used in this study [42].

Additionally, exploratory partial correlation analyses were performed to assess the correlation between the altered functional connectivity and clinical variables, i.e., HDRS score and length in months of the current depressive episode. Age was included as a confounding covariate. Two-tailed levels of significance were set at *p*<0.05 and were uncorrected for multiple comparisons in the correlation analyses [29], [43]. Correlation analyses were performed just within regions previously identified as showing group differences [25], [29]. Using the peak voxels with significant group differences as the centers, the mean z-scores of the spheres including 27 voxels were selected for the correlation analyses.

## Results

### Increased cerebellar functional connectivity in major depression

**Figure 2:**
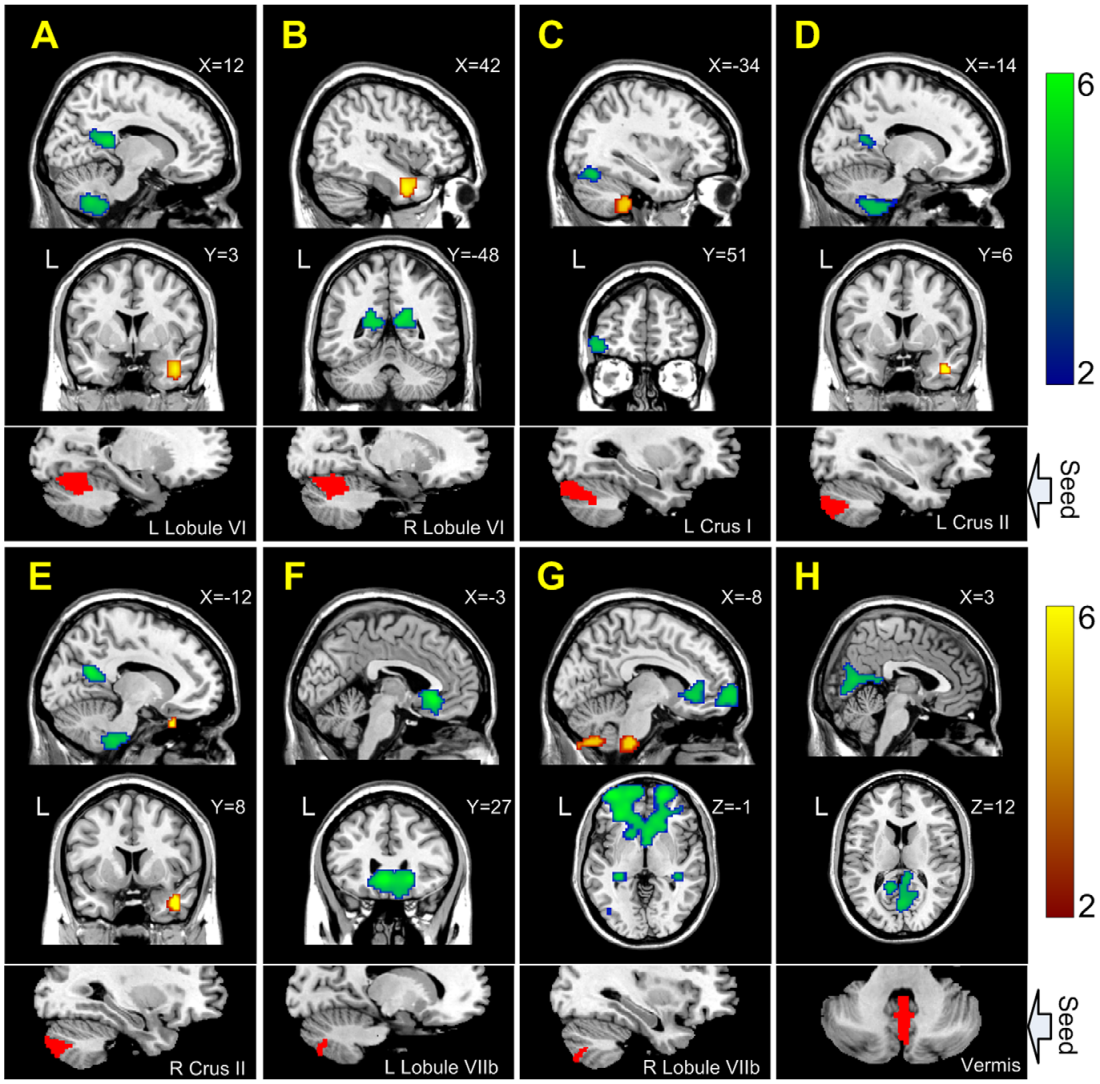
Significantly increased (red) and decreased (blue) connectivity maps of the cerebellar ROIs in the adults with major depression: Lobule VI (A and B), Crus I (C), Crus II (D and E), Lobule VIIb (F and G), and Vermis (H).

**Figure 3:**
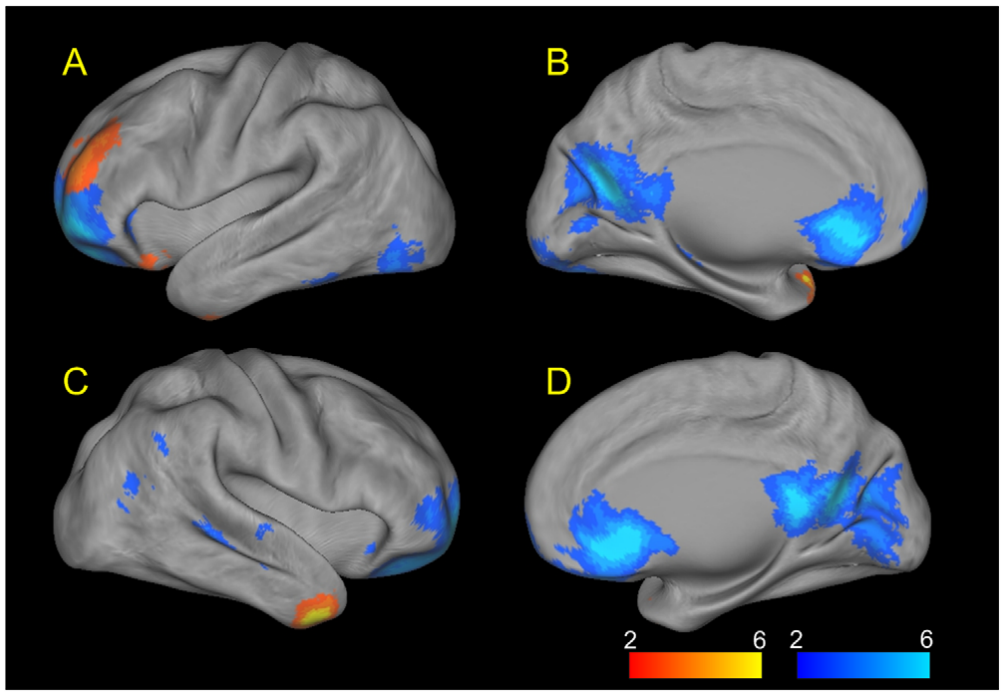
Connectivity maps of significantly increased (red) and decreased (blue) cerebellar-cerebral functional connectivity in the adults with major depression, visualized using CARET 5.62 [58], [59]: (A and B) Left hemisphere, (C and D) Right hemisphere. Relative to the controls, the depressed patients showed a significant connectivity increase between the cerebellum and the temporal poles and left middle frontal cortex as well as a significant connectivity decrease between the cerebellum and the default-mode network, mainly including the ventromedial prefrontal cortex and posterior cingulate cortex/precuneus.

Relative to the healthy controls, the depressed patients showed a significant connectivity increase between the cerebellum (including bilateral Lobule VI and bilateral Crus II) and the temporal poles (BA 21/38) (Table 2 and Figures 2&3), which are key regions in the affective network [28], [44], [45]. Additionally, the functional connectivity between the left Lobule VI and the left middle frontal cortex (BA 10) was enhanced in the depressed patients.

**Table 2:** Brain regions exhibiting significantly increased functional connectivity with the cerebellum in adults with major depression.

Seed Region	Target Area	Side	BA	MNI(x, y, z)			Cluster size	t-score	Mean z(r) (MDD/HC)
L Lobule VI**	Temporal Pole	R	21	42	3	−30	80	5.36	0.09/−0.05
	Middle Frontal Cortex	L	10	−33	54	21	192	4.97	−0.02/−0.14
R Lobule VI**	Temporal Pole	R	38	42	3	−33	91	4.43	0.11/−0.06
L Crus I**	Lobule VIII	L		−39	−39	−48	92	4.66	−0.04/−0.11
R Crus I*	Inferior Temporal Gyrus	R		12	−66	−45	1477	5.26	−0.32/−0.85
L Crus II*	Lobule VIIb/VIII/IX	L/R	38	42	6	−30	18	6.15	0.13/−0.09
	Temporal Pole	R	38	−36	12	−15	16	5.49	0.13/−0.01
R Crus II*	Temporal Pole	L	21	42	6	−30	54	6.14	0.14/−0.11
L Lobule VIIb*	Temporal Pole	R		30	−36	−48	426	5.48	−0.12/−0.37
	Lobule VIII/X	R		−24	−84	−48	248	5.93	−0.19/−0.62
R Lobule VIIb*	Lobule VIII	L		−21	−78	−51	462	5.11	−0.61/−0.90
	Lobule IX	L		9	−69	−48	569	4.99	−0.34/−0.76
	Lobule VIII/IX	R	38	−30	−3	−51	131	5.56	0.01/−0.15
	Superior Temporal Gyrus	L	38	27	0	−54	43	4.50	0.01/−0.04

* *p*<0.05 (False Discovery Rate corrected) with cluster size >10 voxels. ** *p*<0.001 (uncorrected) with cluster size >50 voxels. L: left; R: right; FDR: False Discovery Rate; MDD: Major depressive disorder; HC: Healthy control.

### Decreased cerebellar functional connectivity in major depression

Compared with the healthy controls, the patient group showed a significant connectivity decrease between the cerebellum and brain areas in the DMN (Table 3 and Figures 2&3) [46], [47], [48]. In the depressed patients, functional connectivity with the bilateral Lobule VI, bilateral Crus II, Vermis, and posterior cingulate cortex/precuneus (PCC/PCu) (BA 23/30/31) was reduced. Functional connectivity between the bilateral Lobule VIIb and the ventromedial prefrontal cortex (VMPFC) (BA 11/24/25/32) was also reduced in depressed patients. Additionally, relative to the healthy controls, functional connectivity was decreased between the right Lobule VIIb and the hippocampus in the depressed patients. Moreover, the patient group also showed significantly decreased connectivity between the cerebellum and the ECN (Table 3 and Figures 2&3) [4], [46], [49], i.e., decreased connectivity between the bilateral Lobule VIIb and the superior frontal gyri (BA 9/10), as well as between the left Crus I and the left orbitofrontal gyrus (BA 47).

**Table 3:** Brain regions exhibiting significantly decreased functional connectivity with the cerebellum in adults with major depression.

Seed Region	Target Area	Side	BA	MNI(x, y, z)			Cluster size	t-score	Mean z(r) (MDD/HC)
L Lobule VI**	Posterior Cingulate/Precuneus	L	30/31	18	−45	15	462	5.07	−0.06/0.17
	Lobule VIII/IX	L/R		−9	−48	−51	939	4.92	−0.13/0.14
R Lobule VI*	Posterior Cingulate/Precuneus	R	23/30/31	12	−42	15	200	5.65	−0.12/0.14
	Posterior Cingulate/Precuneus	L	23/30/31	−18	−57	24	164	5.59	0.00/0.21
L Crus I**	Fusiform Gyrus	R	19	−24	−81	−21	144	4.53	−0.27/−0.07
	Orbitofrontal Cortex	L	47	−48	51	−6	57	4.01	−0.16/−0.01
L Crus II**	Posterior Cingulate/Precuneus	L	31	−24	−51	18	78	4.67	−0.03/0.16
	Lobule VIII/IX	L/R		12	−63	−45	771	4.36	−0.23/0.08
R Crus II**	Posterior Cingulate/Precuneus	L	23/31	−12	−57	21	347	4.88	0.09/0.32
	Lobule VIIb/VIII/IX	R		39	−39	−45	754	4.71	−0.06/−0.01
L Lobule VIIb*	Superior Frontal Gyrus	R	10	24	60	6	1363	5.72	0.00/0.28
	Medial Prefrontal Gyrus	L	11/24/25/32	−3	27	−6	438	5.34	−0.12/0.16
	Fusiform Gyrus	L	19	−36	−72	−15	150	4.30	−0.07/0.14
	Superior Temporal Gyrus	R	21/22	69	−12	−3	47	4.23	−0.01/0.06
	Lingual Gyrus	R	18	9	−75	−3	68	4.18	−0.02/0.19
	Crus I	R		54	−45	−30	37	4.28	−0.01/0.08
	Crus I	L		−51	−48	−30	43	4.15	−0.08/0.12
	Supramarginal Gyrus	R	40	54	−45	33	15	3.72	−0.13/−0.05
	Cuneus	L	18	−12	−102	3	12	3.70	0.04/0.11
R Lobule VIIb*	Superior Frontal Gyrus	R	9/10	27	63	6	226	5.63	−0.12/0.20
	Superior Frontal Gyrus	L	9/10	−39	63	−3	548	5.48	−0.04/0.06
	Medial Prefrontal Gyrus	L	11/24/25/32	−21	42	−6	480	5.08	−0.13/0.18
	Hippocampus	L	27	−30	−33	0	45	4.52	−0.02/0.18
	Hippocampus	R	27	39	−33	0	18	4.22	−0.03/0.20
	Crus I	L		−51	−48	−33	57	4.45	−0.09/0.10
	Crus I	R		51	−42	−30	48	4.26	−0.02/0.12
	Inferior Occipital Gyrus	L	19	−39	−72	−12	56	4.13	0.01/0.22
Vermis**	Posterior Cingulate/Precuneus	R	23/31	3	−69	12	334	4.25	−0.21/0.07

* *p*<0.05 (False Discovery Rate corrected) with cluster size >10 voxels. **p<0.001 (uncorrected) with cluster size >50 voxels. L: left; R: right; FDR: False Discovery Rate; MDD: Major depressive disorder; HC: Healthy control.

### Clinical Correlation Analysis

**Figure 4:**
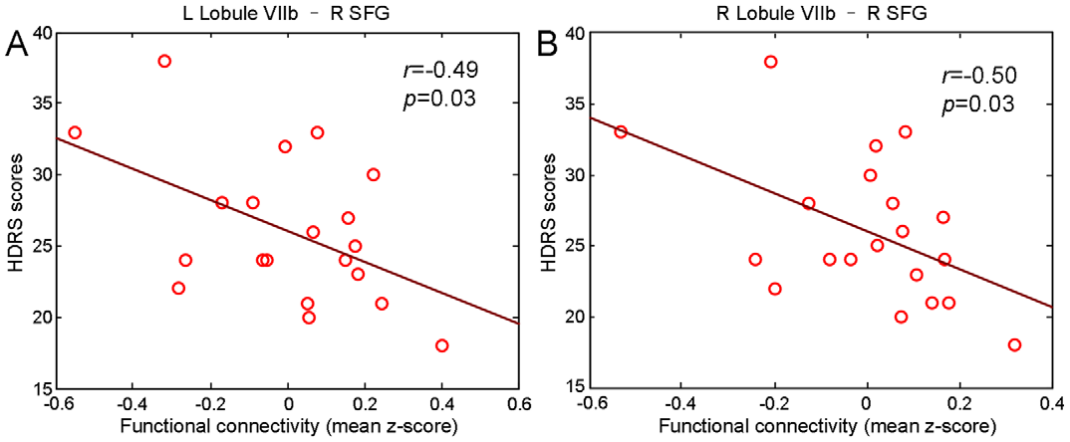
The HDRS scores were significantly negatively correlated with the functional connectivity between the bilateral Lobule VIIb and right superior frontal gyrus in the depressed patients. HDRS: Hamilton Depression Rating Scale; SFG: superior frontal gyrus; L: left; R: right.

Depression refractoriness, as measured by the HDRS score, was negatively correlated with the functional connectivity between the bilateral Lobule VIIb and the right superior frontal gyrus, with correlation values of −0.49 (left, *p* = 0.03) and −0.50 (right, *p* = 0.03) at the trend level, respectively (Figure 4). No regions had significant associations with the length in months of the current depressive episode.

## Discussion

Using a seed-based method, our study demonstrated that functional connectivity between the cerebellum and intrinsic connectivity networks was significantly altered in adults with major depression during rest. In particular, significantly increased functional connectivity was observed between the cerebellum and the temporal poles and left middle frontal cortex in patients with major depression, while significantly decreased connectivity between the cerebellum and the regions in the DMN and ECN was also found. It has not been commonly reported that the cerebellum contributes to intrinsic connectivity network alterations in adults with major depression. Furthermore, the HDRS score was negatively correlated with the functional connectivity between the bilateral Lobule VIIb and the right superior frontal gyrus in depressed patients.

In the first finding, the current study identified increased positive (left Crus I and bilateral Crus II) or negative (right Lobule VIIb) resting-state functional connectivity between the cerebellum and the temporal poles during depression. Temporal poles, which are key regions in the affective network [28], [42], [43], have a role in both social and emotional processes, including face recognition and theory of mind [45]. Abnormal connectivity between the temporal poles and the cerebellum may reflect dysfunctions of visceral emotional monitoring, which is compromised in depression [27].

In the second principal finding, adults with depression, compared with healthy controls, demonstrated decreased functional connectivity between the cerebellum (primarily including the right Lobule VI, left Crus I, bilateral Lobule VIIb, and Vermis) and the DMN (mainly including the PCC/PCu, VMPFC, and hippocampus) [27], [47], [48], [50]. The PCC/PCu is a key region for sustaining self-processing during rest [51]. The VMPFC has been associated with emotional evaluation and regulation and reward processing [52], [53]. The DMN, which is known to be involved in self-referential activity, episodic memory retrieval, and emotion modulation [47], [48], [54], [55], has been documented to be dysfunctional in depression [25], [27], [48]. Recent rs-fcMRI studies in healthy subjects indicated that the Lobule VIIb participated in the DMN [4], [6], [7]. In this study, lower connectivity between the Lobule VIIb and the regions in the DMN was observed. The cerebellar lesions have been suggested to induce cognitive affective syndrome [10]. Accordingly, abnormal resting-state functional connectivity between the cerebellum and the DMN may not only account for disturbances in emotional behavior and other cognitive aspects of depressive syndrome in depressed patients [25], [27], [48], but may also demonstrate the higher-order functions of the cerebellum.

Finally, it was demonstrated that connectivity between the cerebellum (containing the left Crus I and the bilateral Lobule VIIb) and the ECN was decreased in adults with major depression. The ECN, mainly including the superior frontal gyri and orbitofrontal cortex [4], [44], [47], is involved in cognitive control and decision-making and is known to be impaired in depression [27]. Previous studies have shown cerebellar regions participating in the ECN in healthy controls [4]. In the current study, decreased negative connectivity between the left Crus I and the left orbitofrontal cortex, and decreased positive or negative connectivity between the bilateral Lobule VIIb and the superior frontal gyri, was observed in major depression. Moreover, the HDRS score was negatively correlated with the functional connectivity between the bilateral Lobule VIIb and the right superior frontal gyrus in depressed patients. Patients with cerebellar lesions exhibit deficits in planning, executive control, memory and learning, and attention processing [56]. We speculate that decreased the functional connectivity between the cerebellum and the regions in the ECN during rest may subserve the cognitive control function deficits encountered in depression [57].

In addition, it should be noted that the abnormalities in the cerebellar-cerebral functional connectivity exhibited hemispheric differences in major depression, mainly including the altered functional connectivity of the bilateral Lobule VIIb, bilateral Lobule VI, and bilateral Crus I. These results may be in accordance with previous studies demonstrating structural or functional asymmetries in the cerebellum in healthy subjects and psychiatric patients [5].

There were multiple limitations in our study related to sample size, scanner variability, and the lack of a large independent dataset to confirm our findings and to further improve our understanding of the affective and cognitive functions of the cerebellum in models of the pathophysiology of depression. Datasets with a lager sample size could enhance the correlation analyses between functional connectivity coefficients and clinical variables. Additionally, though we have used some denoising methods to reduce the negative impacts of those artifacts related to motion and physiological sources in this study, some potential confounding factors (such as greater head movement in the patient group) should be avoided in future studies. Besides, some statistical maps that did not meet the combined threshold of *p*<0.05 (False Discovery Rate corrected) with cluster size >10 voxels but met a relaxed threshold of *p*<0.001 (uncorrected) with cluster size >50 voxels were also reported in this study [42]. Though these results may be a little exploratory, but all these results would still be valuable to lighten the pathological mechanism of major depression.

In summary, this study demonstrates the significantly altered functional connectivity between the cerebellum and intrinsic connectivity networks in adults with major depression, including increased cerebellar coupling with the affective network and reduced coupling with the DMN and ECN. The cerebellar connectivity network alterations may be associated with emotional and cognitive impairments in major depression. These findings may also provide further evidence for the considerable role of the cerebellum in the pathophysiological models of depression.
